# Serum Granzyme B in Parkinson’s Disease: A Case–Control Study of Diagnostic Association

**DOI:** 10.3390/jcm15187115

**Published:** 2026-09-14

**Authors:** Marwa Fareed Almulhim, Shimaa Elgamal, Amany A. Ghazy, Sally A. Saleh, Samar A. Eissa, Salma A. Shatara, Youssef A. Shatara, Eman K. Rashwan, Moamen Abdelfadil Ismail

**Affiliations:** 1 Internal Medicine Department, College of Medicine, Jouf University, Sakaka 72388, Saudi Arabia; mfalmulhim@ju.edu.sa; 2 Neuropsychiatry Department, Faculty of Medicine, Kafrelsheikh University, Kafrelsheikh 33516, Egypt; shimaa_elgamal@med.kfs.edu.eg; 3 Microbiology and Immunology Unit, Department of Pathology, College of Medicine, Jouf University, Sakaka 72388, Saudi Arabia; 4 Medical Microbiology & Immunology Department, Faculty of Medicine, Kafrelsheikh University, Kafr El Sheikh 35111, Egypt; sally_saleh2014@med.kfs.edu.eg (S.A.S.); asamar74@med.kfs.edu.eg (S.A.E.); 5 Faculty of Medicine, Kafrelsheikh University, Kafr El Sheikh 35111, Egypt; salma_1222a@med.kfs.edu.eg; 6 Faculty of Medicine, Alexandria National University, Alexandria 21526, Egypt; 2301055@anu.edu.eg; 7 Department of Physiology, College of Medicine, Jouf University, Sakaka 42421, Saudi Arabia; krashwan@ju.edu.sa; 8 Department of Physiology, Faculty of Medicine, Al-Azhar University, Assuit 71524, Egypt; 9 Department of Internal Medicine, Faculty of Medicine, Capital University, Cairo 11795, Egypt; moamen.fadil83@gmail.com

**Keywords:** Parkinson’s disease, granzyme B, biological indicator, diagnostic association, case–control study

## Abstract

**Background/Objectives:** Parkinson’s disease (PD) is a neurodegenerative disease with a heterogeneous nature. Many molecular pathways are engaged in PD pathogenesis. Granzyme B (GrB) is an enzyme released by CTLs and has roles in neuroinflammation, axonal degeneration, demyelination, and neuronal ischemic death. However, little is reported about its role in PD pathogenesis and deterioration. To evaluate the role of GrB in PD. **Method:** A total of 94 participants were recruited from outpatient neurology clinics (47 PD patients and 47 healthy controls). Serum GrB levels were measured using ELISA. **Results:** Among PD patients, the age of onset was 57.30 ± 4.92 years, and the duration of illness was 7.13 ± 4.20 years. Sociodemographic data revealed statistically significant associations between the development of PD and HCV infection (0.001*), family history of neuropsychiatric illness (0.004*), and/or PD (0.028*). MoCA TS showed a significant reduction in cognitive performance among PD patients even after correction (*p* < 0.001*). H_Y score showed that >50% of PD patients were clustered in stages 1 and 2, and motor scores showed a substantial reduction. GrB levels were markedly increased among PD patients compared with the control group (1721.4 ± 588.8 pg/mL vs. 418.4 ± 131.3 pg/mL) (*p* < 0.001*). This indicates a strong association between elevated granzyme B and PD. However, no statistically significant correlations were observed between GrB levels and the parameters studied. **Conclusion:** GrB levels are significantly elevated among PD patients. This reflects underlying immune activation or inflammatory processes associated with the progression of PD. GrB showed a promising diagnostic association with PD, but was not correlated with disease severity, activity, or duration in this cohort. Formal evaluation of GrB’s diagnostic accuracy in an independent, disease-control-inclusive cohort is warranted before it can be considered a diagnostic biomarker.

## 1. Introduction

Parkinson’s disease (PD) is a neurodegenerative disease characterized by progressive bradykinesia (slowness of movement), rigidity, resting tremor, and postural unsteadiness [1]. It was first described in 1817 as tremulous, involuntary shaking with diminished muscular power and an unassailable propensity to run when wishing only to walk [2].

PD is ranked as the 2nd most common neurodegenerative disorder, affecting 2–3% of people older than 65 years. It is caused by dopamine deficiency and intracellular α-synuclein aggregates, which result from degeneration of nigral dopaminergic neurons (DNs) and neuronal loss [3,4].

The challenge of diagnosing Parkinson’s disease lies in its heterogeneous nature with a broad disease spectrum (motor and non-motor symptoms). Symptoms including cognitive decline, mood disorders, and sleep disturbances can occur at an early stage of PD, resulting in disturbed quality of life [5]. Beyond neuroinflammation, multiple pathways and mechanisms contribute to its molecular pathogenesis, including mitochondrial dysfunction, calcium dysregulation, oxidative stress, and impaired axonal transport [3].

Recent advancements in understanding the pathophysiology of PD, including the role of genetic factors and environmental influences, have provided insights that could lead to earlier diagnosis and more tailored treatment options. As awareness of Parkinson’s disease grows, it is crucial to recognize the diverse manifestations of this condition, which can vary significantly among individuals [6].

Granzyme B (GrB) is a serine protease that plays a crucial role in the induction of apoptosis (programmed cell death). While GrB is primarily associated with the cytotoxic function of T lymphocytes and natural killer cells, emerging evidence suggests its involvement in the pathogenesis of various neurological diseases [7,8].

It was reported that this cytotoxic proteinase can induce perforin-independent neurotoxicity by interacting with the membrane-bound Gi-coupled Protease-activated receptor-1 (PAR-1), leading to activation of Voltage-gated Potassium Channel 1.3 (Kv1.3) and Notch-1 [8].

GrB can contribute to many inflammatory conditions by cleaving cytokines, cell receptors, and the extracellular matrix. It regulates various biological processes that are crucial in the occurrence, severity, and/or progression of inflammatory diseases and cancers [9,10,11]. Elevated levels of GrB have been observed in many cardiac, pulmonary, inflammatory, and carcinogenic diseases [12,13,14]. Targeting GrB may inhibit pathological conditions and provide promise for treatment, as reported in the case of urinary bladder and pancreatic carcinoma [15].

In addition, it was reported that GrB can cause central nervous system axonal degeneration and diabetic cognitive dysfunction. Inhibition of GrB resulted in decreased demyelination, endoplasmic reticulum stress, and amelioration of the cognitive dysfunction in diabetic mice [16]. However, limited research has been done regarding its role in Parkinson’s disease.

Thus, the case–control study assessed serum granzyme B levels among PD patients and healthy controls. This was done to evaluate its role in PD activity and severity.

## 2. Materials and Methods

### 2.1. Ethical Approval

The study protocol has been approved by the Scientific Research Ethics Committee of Kafrelsheikh University, Egypt (KFSIRB200-413 on 30 September 2024). The study followed the ethical guidelines of the 1975 Declaration of Helsinki without any risk to the participants. All participants signed informed written consent forms. As an observational, non-interventional case–control study, it was not registered in a clinical trial registry. The study is reported in accordance with the STROBE (Strengthening the Reporting of Observational Studies in Epidemiology) guidelines for case–control studies.

### 2.2. Study Design and Sample Size

The current case–control study enrolled 47 PD patients and 47 age- and gender-matched healthy controls. Sample size was determined pragmatically based on outpatient clinic accrual over the study period; no formal a priori power calculation was performed. Participants were recruited from outpatient neurology clinics. Informed consent was obtained from all participants.

Inclusion Criteria: Patients aged 45–85 who have been diagnosed with Parkinson’s disease by a movement-disorder-trained neurologist according to the UK Parkinson’s Disease Society Brain Bank clinical diagnostic criteria (bradykinesia plus at least one of rigidity, resting tremor, or postural instability, with supportive criteria and exclusion of atypical features).

Exclusion Criteria: Patients with other neurodegenerative diseases, recent infections, or inflammatory conditions were excluded from the study. Patients with atypical parkinsonian syndromes (e.g., progressive supranuclear palsy, multiple system atrophy, corticobasal syndrome) or drug-induced/vascular parkinsonism were also excluded.

### 2.3. Data and Sample Collection

Blood samples (10 mL) were collected via venepuncture, and serum was isolated by centrifugation at 1500× *g* for 10 min at 4 °C, then stored at −80 °C until analysis. Serum granzyme B levels were measured using ELISA kits specific for human granzyme B. Triplicates for each sample and standard controls were used in each assay.

Patients were evaluated clinically using the Montreal Cognitive Assessment (MoCA), Hoehn and Yahr (H_Y) staging, and the Unified Parkinson’s Disease Rating Scale, Part III (UPDRS-III) motor scale to determine severity. UPDRS-III and H_Y staging were each scored twice: once after overnight withdrawal of antiparkinsonian medication (practically defined OFF, ≥12 h since last dose) and again 60–90 min after the patient’s usual morning levodopa dose (ON). Levodopa-equivalent daily dose and treatment duration at the time of assessment were not systematically recorded for all participants. Demographic data included age, gender, onset of disease, family history, HCV infection, and comorbidities.

Blood samples (10 mL) were collected via venepuncture, prior to the day’s antiparkinsonian medication dose (OFF state), and serum was isolated by centrifugation at 1500 *g* for 10 min at 4 °C, then stored at −80 °C until analysis. Serum granzyme B levels were measured using ELISA kits specific for human granzyme B (Human Granzyme B ELISA Kit, Cat # BMS2027-2, Invitrogen, ThermoFisher Scientific, Waltham, MA, USA). Triplicates of each sample and standard controls were used.

### 2.4. Statistical Analysis of the Data

Data were analyzed using IBM SPSS software package version 27.0. (Armonk, NY, USA: IBM Corp, released in 2020). Categorical data were summarized as numbers and percentages. Chi-square test was applied to compare the two groups. Alternatively, Fisher’s Exact and Monte Carlo correction tests were applied when more than 20% of the cells had expected counts less than 5. Continuous data were tested for normality by the Shapiro–Wilk test for ≤50 cases. Quantitative data were expressed as range (minimum and maximum), mean, standard deviation, median, and interquartile range (IQR) for normally distributed quantitative variables. Student’s *t*-test was used to compare two groups. For non-normally distributed quantitative variables, the Mann–Whitney test was used to compare two groups. Significance of the obtained results was judged at the 5% level. Association tests against serum GrB level were done and a Bonferroni correction was additionally applied (corrected significance threshold α = 0.05/28 ≈ 0.0018); both uncorrected and Bonferroni-corrected *p*-values are reported for these exploratory analyses.

## 3. Results

### 3.1. Participants’ Demographic Data

The current study enrolled 94 participants (47 patients and 47 healthy controls). The age of onset (AOO) of PD ranged between 50.0 and 71.0 years (Mean ± SD was 57.30 ± 4.92), and the duration of illness (DOI) was 2.0–22.0 years (Mean ± SD was 7.13 ± 4.20) (Table 1).

There was a statistically significant relationship between PD and HCV infection (0.001*), family history of neuropsychiatric illness (0.004*), and family history of Parkinson’s disease (0.028*). Note that current age at enrolment (Table 1) and age of onset (age at first symptom, above) are distinct variables and are not expected to be numerically identical.

### 3.2. Treatments Used for Parkinson’s Patients

Regarding the treatment used for Parkinson’s patients, more than 50% of patients were using Levodopa, which acts as the primary source of dopamine to improve motor and cognitive function. Other drugs are illustrated in Table 2.

### 3.3. Montreal Cognitive Assessment (MoCA) Total Scores of the Studied Participants

MoCA is a screening tool used to detect mild cognitive impairment and can be completed in about 10 minutes. It evaluates multiple domains, including short-term memory, attention, concentration, working memory, executive function, visuospatial skills, language, and orientation [17]. MoCA provides a comprehensive test of executive and frontal lobe functions [18].

MoCA TS ranged from 18.0 to 29.0 among PD patients, and from 24.0 to 29.0 among the control group. This indicates a marked, statistically significant reduction in cognitive performance among patients compared with controls. These lower scores remained in the corrected MoCA (*p* < 0.001* for both comparisons) (Table 3).

### 3.4. Hoehn and Yahr (H_Y) Scale of the Studied Participants

The H_Y scale, introduced in 1967, is a widely used clinical tool for staging the severity and progression of PD. It classifies patients into five stages based on motor symptoms, such as tremor, rigidity, and postural instability. The scale is frequently used because it helps monitor disease progression and assess functional disability [19].

Among PD patients, a predominance of mild to moderate disease severity was observed, as more than half of PD patients were clustered in stages 1 and 2 (in both OFF and ON states). Motor scores showed a substantial reduction in symptom severity in the ON state (mean 37.64 ± 12.05) compared to the OFF state (mean 64.11 ± 14.11) (Table 4).

### 3.5. Serum Levels of Granzyme B Among the Studied Participants

**Figure 1:**
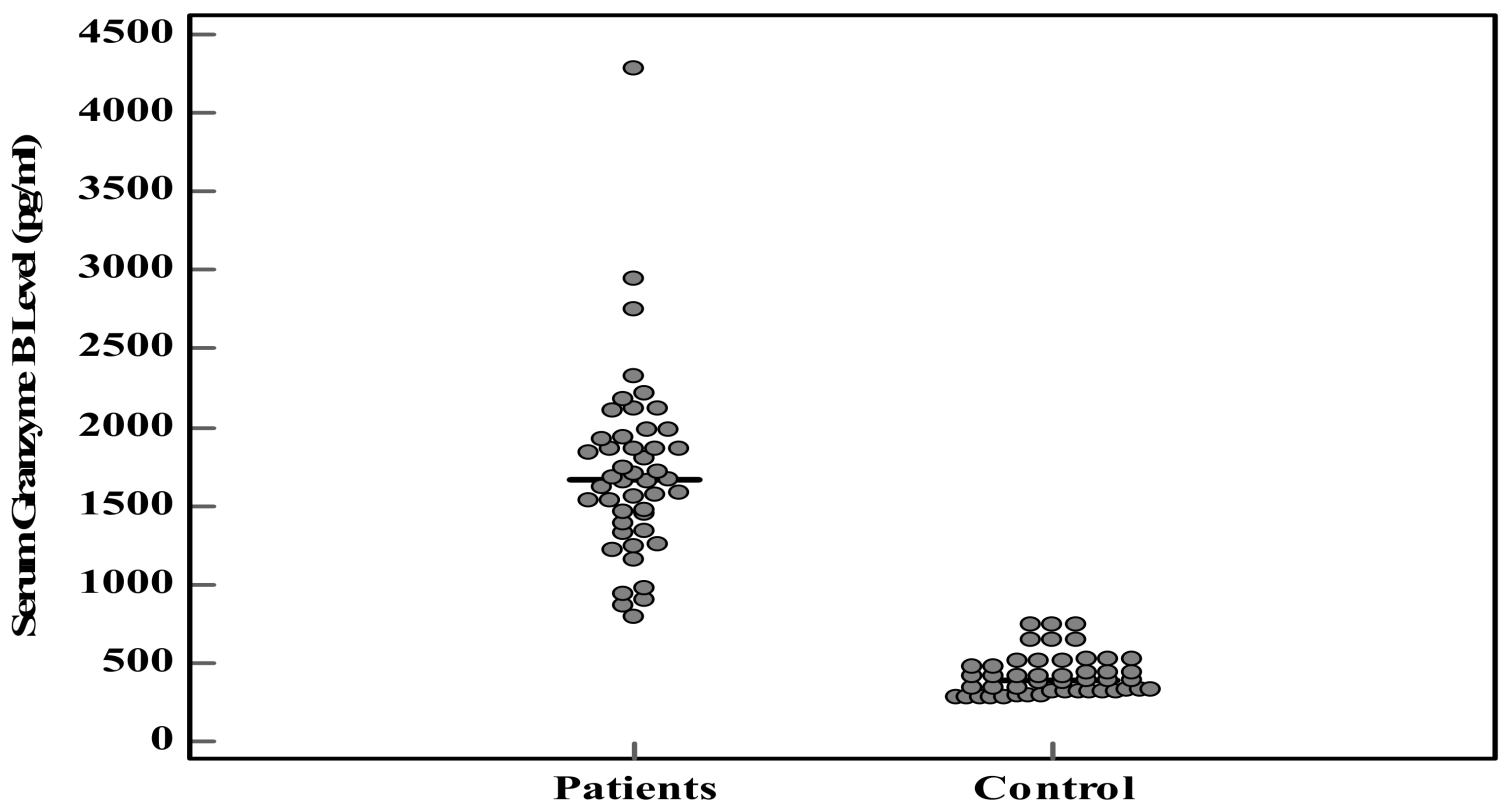
Comparison between the two groups studied according to serum granzyme B levels (pg/mL).

Measuring GrB levels revealed a marked increase among PD patients compared with the control group (1721.4 ± 588.8 pg/mL vs. 418.4 ± 131.3 pg/mL) (*p* < 0.001*). This indicates a strong association between elevated granzyme B and PD (Table 5 and Figure 1).

### 3.6. Correlation Analysis

Correlation analysis between GrB levels and the studied parameters in the patient group was performed using Spearman’s coefficient, Mann–Whitney test, and Kruskal–Wallis test. No statistically significant correlations were observed (Table 6 and Table 7). After Bonferroni correction for multiple testing across the 28 tests reported in Table 6 and Table 7, none of the relations remained statistically significant, including the borderline uncorrected association observed with smoking status (uncorrected *p* = 0.027; Bonferroni-corrected *p* = 0.756).

## 4. Discussion

PD is the second most prevalent age-related neurodegenerative disorder that considerably impairs day-to-day functioning [20]. The core feature of PD is bradykinesia combined with either resting tremor or stiffness [21]. Furthermore, falls are a frequent and dangerous side effect of neurological disorders [22].

Dopamine plays a key role in controlling movement. As DNs deteriorate, the neural circuits connecting the basal ganglia and motor cortical areas become disrupted. This leads to characteristic motor impairments that significantly affect a patient’s daily activities and overall quality of life [23]. The progressive loss of DNs, dopamine oxidation-mediated neurotoxicity, and associated dopamine depletion are hallmarks of Parkinson’s disease. This was proposed to be mediated by autophagy-lysosome pathway (ALP) dysfunction, increasing dopamine transporter (DAT) density per neuron, and enhancing dopamine re-uptake, oxidation, and loss of DNs [24].

Granzymes have cytotoxic and/or proinflammatory properties, especially GrB, which is also located in the extracellular space, causing cleavage of adhesion molecules, receptors, cytokines, autoantigens, and chronic tissue injury. This has been found in many age-related diseases, including Parkinson’s disease [25]. High levels of GrB were reported in the substantia nigra (SN) of PD patients. This suggests its involvement in dopaminergic degeneration, inflammation, and microglial activation. Richardson KC et al. (2024) reported that genetic deletion and/or pharmacological suppression of granzyme B decreases the premature aging and/or disease phenotypes in animal models [25].

The current study aimed to assess serum granzyme B levels in PD to evaluate its role in both disease activity and severity. The mean ± SD of AOO and DOI among PD patients were 57.30 ± 4.92 years and 7.13 ± 4.20 years, respectively. There was a statistically significant relationship between Parkinson’s disease and HCV infection (0.001*), family history of neuropsychiatric illness (0.004*), and family history of PD (0.028*).

In previous studies, a statistically significant association between PD development and HCV infection was reported [26,27,28]. Tsai HH et al. (2016) collected data on 49,967 patients with viral hepatitis from the Taiwan National Health Insurance Research Database between 2000 and 2010 [26]. They found a high incidence of PD among HCV-infected patients [26]. Wijarnpreecha K et al. (2018) conducted a meta-analysis to summarize all available studies that reported the risk of Parkinson’s disease among HCV-infected patients [27]. They observed a higher risk of PD among patients with chronic HCV infection [27]. Choi HY et al. (2020) conducted a population-based prospective study to determine if there is a relation between viral infection and Parkinson’s disease among Korean individuals [28]. They found that HBV and HCV-infected patients older than 40 years are at high risk of developing PD and should be monitored for early intervention [28]. All these findings suggest a possible role of chronic inflammation or viral neurotropic mechanisms in the pathogenesis of PD.

Within the current study, GrB levels did not differ significantly between HCV-positive and HCV-negative patients (Table 7; *p* = 0.542), arguing against active HCV infection as the principal driver of the GrB elevation observed in PD. However, HCV prevalence was markedly higher among patients (25.5%) than controls (2.1%), so residual confounding by HCV-related immune activation cannot be fully excluded. This should be addressed directly in future HCV-stratified or HCV-excluded cohorts.

Consistently, the significant association observed between PD and a positive family history of Parkinson’s disease aligns with the study published by Liu X et al. (2018), who reported that the prevalence of PD is 0.52% among the first-degree relatives of PD patients [29]. The relative risk (RR) was highest among twins, followed by siblings, offspring, parents, and spouses, respectively [29]. Also, Torti M et al. (2020) reported a higher risk of PD among elderly males and first-degree relatives [30]. These results, together with the current study findings, reflect the major contribution of genetic susceptibility to PD.

Furthermore, the significant association between PD and the family history of neuropsychiatric illness in the current study is supported by research reporting common family aggregation, genetic, and environmental risk factors between PD and neuropsychiatric disorders [29,31]. All these findings support the concept of overlapping pathways and a multifactorial etiology of PD, involving infectious, genetic, and neuropsychiatric components.

Regarding the PD scoring used in the current study, MoCA total and corrected scores showed mild cognitive impairment among PD patients, with a mean MoCA total score of 24.21 ± 2.63 compared to 26.38 ± 1.91 in controls. This difference remained after correction (24.68 ± 2.62 vs. 26.72 ± 2.01), confirming that this deficit is robust and not attributable to confounding factors addressed by the correction and indicating significant cognitive dysfunction among PD patients compared to healthy controls. These findings provide further confirmation of cognitive impairment in PD reported in previous studies [17,32,33,34].

At the brain level, Li QQ et al. (2022) investigated the effects of white matter structural damage and cognitive impairment in PD patients using diffusion tensor imaging (DTI) [35]. They found a correlation between white matter damage, areas with decreased MoCA visual scores, and the severity of PD [35].

Another hallmark of disease progression and treatment responsiveness is the H_Y stage and motor scores.

Although Parkinson’s disease has been extensively investigated, the gold standard diagnostic and monitoring scales —namely the Movement Disorder Society-Unified Parkinson’s Disease Rating Scale (MDS-UPDRS) and the Hoehn and Yahr (H-Y) scale—rely solely on clinical and subjective evaluations, with no significant laboratory parameter [36,37]. The MDS-UPDRS offers a standardized motor and non-motor assessment; it depends heavily on the observational judgment of the evaluating clinician [38]. Both the MDS-UPDRS and H-Y scoring systems group the motor patterns into numeric values that can mask continuous, minor changes in disease severity, especially during the early progressing stages [37]. These gold-standard scales assess only static cross-sectional symptoms during a short clinic visit; thus, they are unable to measure different, day-to-day symptom variability, transient off-medication periods, or transient stress effects that the patients are exposed to in their daily lives [38].

Results of the current study reflect a predominance of mild and moderate disease severity, where more than 50% of PD patients clustered in H_Y stages 1 and 2. A clear improvement was observed in the ON state of H-Y, where a higher proportion of patients were in lower stages (e.g., stage 1 increased from 21.3% to 36.2%, while stages 3 and 4 markedly decreased). Similarly, motor scores showed a substantial reduction in symptom severity in the ON state (37.64 ± 12.05) compared to the OFF state (64.11 ± 14.11). This reflects a favorable response to the treatment used; more than half of the patients were using Levodopa. These findings are in line with established research demonstrating that Levodopa improves objective rigidity in PD by reducing its biomechanical neural component and long-latency stretch reflexes (LLRs) [39]. Morover, it was reported that patients with early PD may develop a long-duration response (LDR) to L-DOPA [40].

Over two centuries, the diagnosis of PD has remained challenging and is based only on characteristic clinical features and neurological examination. It is distinguished from other movement disorders via genetic testing and ancillary investigations such as olfactory testing, MRI, and a few potential cerebrospinal fluid biomarkers [41,42]. A significant response to dopaminergic therapy remains a key supportive feature. PD risk and progression are linked to integrating metabolic and frailty assessment (e.g., TyGFI), and reduced T-cell–mediated immunity, as supported by multiple clinical studies [43,44,45].

Cytotoxic effector T-lymphocytes (CTLs) were associated with elevated expression of multiple genes related to cytotoxicity, including GrB, in individuals with PD [46].

In the current study, measuring GrB levels revealed a marked increase among PD patients compared with the control group (1721.4 ± 588.8 pg/mL vs. 418.4 ± 131.3 pg/mL) (*p* < 0.001*). This indicates a strong association between elevated granzyme B and PD, which is supported by the extremely low U value, reinforcing the robustness of the observed difference. This also reflects the underlying activation of the immune system, particularly the CTLs that secrete GrB.

These results agree with Galiano-Landeira J et al. (2020), who reported that CTLs containing GrB granules invade the SN with other CTLs that express GrA and K, and/or IFNγ at the early stage of PD [47]. Furthermore, it was reported that GrB, a cytotoxic protease secreted by CTLs, inhibits neuronal differentiation and the proliferation of neural progenitor cells (NPCs) via activation of a Gi-protein-coupled receptor, resulting in decreased intracellular cAMP and expression of Kv1.3 [48].

Another experimental study confirmed GrB’s roles in reducing neuronal viability and inducing neurotoxicity in a dose-dependent manner [49]. Groh J et al. (2021) [50] and Latour YL et al. (2026) [51] reported that CD8+ CTLs accumulate and damage neurons through GrB activity.

In addition, Gate D et al. (2020) have identified GrB-expressing CTLs in proximity to dopaminergic neurons (DNs) in PD patients [52]. The same was reported by Capelle CM et al. (2023), who observed a high frequency of enhanced GrB-expressing CTLs in the brains of patients with idiopathic PD [53]. These cells infiltrate the SN and contact DNs at an early premotor stage of PD, resulting in α-synuclein aggregation and neuronal loss [54].

Thus, our findings support a diagnostic association between elevated serum GrB and PD. Whether GrB reflects or predicts disease progression or severity remains an open question that this cross-sectional, single-time-point design cannot address, and the immune-activation interpretation discussed above should be regarded as a biologically plausible hypothesis rather than a conclusion demonstrated by our data. Further mechanistic research is needed to clarify how GrB contributes to neuronal injury in PD and whether it represents a viable therapeutic target.

The main limitation of the current study is the small sample size because no formal a priori sample-size calculation was performed, and several subgroup analyses (Table 7) rest on small cells (*n* = 4–9), limiting statistical power; these should be read as exploratory. The study lacks a disease-control group (e.g., other parkinsonian syndromes), so we cannot claim that GrB elevation is specific to PD rather than a feature shared with other neurodegenerative conditions. Furthermore, chronic HCV infection was more prevalent among patients than controls, and although GrB did not differ significantly by HCV status within the PD group, residual confounding cannot be excluded. Finally, levodopa-equivalent daily dose (LEDD) was not systematically calculated for this cohort, and treatment duration was unrecorded, even though both could influence the magnitude of the ON–OFF motor difference reported in Table 4.

## 5. Conclusions

GrB levels are significantly elevated among PD patients. There was a strong diagnostic association between elevated granzyme B and PD. This elevation is consistent with, but does not by itself demonstrate, underlying immune activation associated with PD; GrB was not correlated with disease severity, activity, or duration in this cohort. Formal assessment of its diagnostic accuracy and evaluation in a disease-control and longitudinal design is warranted.

## Figures and Tables

**Table 1:** Sociodemographic and clinical data of the participants.

	Patients (*n* = 47)	Control (*n* = 47)	Test of Sig.	*p*
Gender				
Male	36 (76.6%)	41 (87.2%)	χ^2^ = 1.795	0.180
Female	11 (23.4%)	6 (12.8%)		
Age (years)				
Min.–Max.	47.0–83.0	56.0–84.0	t = 1.609	0.112
Mean ± SD	60.04 ± 8.50	62.43 ± 5.55		
Work				
Non-working	8 (17.0%)	4 (8.5%)	χ^2^ = 3.137	0.208
Working	19 (40.4%)	15 (31.9%)		
Retired	20 (42.6%)	28 (59.6%)		
Smoking				
Non-smoker	19 (41.3%)	18 (38.3%)	χ^2^ = 1.853	0.396
Smoker	16 (34.8%)	22 (46.8%)		
Ex-Smoker	11 (23.9%)	7 (14.9%)		
Diabetes Mellitus	15 (31.9%)	16 (34.0%)	χ^2^ = 0.048	0.826
Hypertension	21 (44.7%)	18 (38.3%)	χ^2^ = 0.394	0.530
Hepatitis C virus	12 (25.5%)	1 (2.1%)	χ^2^ = 10.802 *	0.001 *
Isolated Systolic Hypertension	4 (8.5%)	5 (10.6%)	χ^2^ = 0.123	*p*_FE_ = 1.000
Hyperlipidaemia	11 (23.4%)	16 (34.0%)	χ^2^ = 1.299	0.254
Consanguinity	12 (25.5%)	6 (12.8%)	χ^2^ = 2.474	0.116
Family history of neuropsychiatric illness	10 (21.3%)	1 (2.1%)	χ^2^ = 8.340 *	0.004 *
Family history of Parkinson’s	12 (25.5%)	4 (8.5%)	χ^2^ = 4.821 *	0.028 *

t: Student’s *t*-test, χ^2^: Chi-square test, FE: Fisher Exact test, *p*: *p*-value for comparing between the two studied groups, *: Statistically significant at *p* ≤ 0.05.

**Table 2:** Distribution of cases studied according to drug in the patient group (*n* = 47).

	No. (%)
Drug	
Levodopa	24 (51.1%)
DOPA agonists	13 (27.7%)
Amantadine	20 (42.6%)
Other treat	33 (70.2%)

DOPA agonists: dopamine agonists.

**Table 3:** Comparison between the two groups studied according to MOCA.

	Patients (*n* = 47)	Control (*n* = 47)	t	*p*
MoCA TS				
Min.–Max.	18.0–29.0	24.0–29.0	4.582 *	<0.001 *
Mean ± SD.	24.21 ± 2.63	26.38 ± 1.91		
Median (IQR)	24.0 (22.0–26.50)	26.0 (25.0–28.0)		
MoCA corrected				
Min.–Max.	19.0–30.0	24.0–30.0	4.240 *	<0.001 *
Mean ± SD	24.68 ± 2.62	26.72 ± 2.01		
Median (IQR)	25.0 (22.50–27.0)	27.0 (25.0–29.0)		

MoCA TS: Montreal Cognitive Assessment Total Score, IQR: Interquartile range, SD: Standard deviation, t: Student’s *t*-test, *p*: *p*-value for comparing between the two studied groups. *: Statistically significant at *p* ≤ 0.05. The MoCA-corrected score adds one point for participants with ≤12 years of education, per the standard MoCA education-correction rule. This is why its range/mean can exceed the uncorrected total score, including reaching the ceiling of 30.

**Table 4:** Descriptive analysis of the cases studied according to H_Y and motor score in the patient group (*n* = 47).

	OFF	ON
H_Y		
0	0 (0.0%)	3 (6.4%)
1	10 (21.3%)	17 (36.2%)
2	21 (44.7%)	23 (48.9%)
3	13 (27.7%)	4 (8.5%)
4	3 (6.4%)	0 (0.0%)
Motor score		
Min.–Max.	32.0–116.0	20.0–85.0
Mean ± SD	64.11 ± 14.11	37.64 ± 12.05
Median (IQR)	65.0 (55.0–73.0)	35.0 (29.0–46.0)

H_Y: Hoehn and Yahr stage, which is a scale used to describe the severity and progression of Parkinson’s disease; IQR: Interquartile range; SD: Standard deviation.

**Table 5:** Serum granzyme B levels (pg/mL) among participants.

Serum Granzyme B Level (pg/mL)	Patients (*n* = 47)	Control (*n* = 47)	U	*p*
Min.–Max.	792.3–4278.4	275.3–745.6	0.000 *	<0.001 *
Mean ± SD	1721.4 ± 588.8	418.4 ± 131.3		
Median (IQR)	1666.4 (1417.2–1932.7)	394.6 (312.6–495.1)		

IQR: Interquartile range, SD: Standard deviation, U: Mann–Whitney test, *p*: *p*-value for comparison between the two studied groups, *: Statistically significant at *p* ≤ 0.05.

**Table 6:** Correlation between serum granzyme B level and different parameters in patient group (*n* = 47).

Serum GrB Level (pg/mL) vs.	r_s_	*p*	*p* (Bonferroni)
MOCA TS	0.099	0.507	1.000
MOCA corrected	0.062	0.681	1.000
Motor OFF	−0.033	0.826	1.000
Motor ON	−0.141	0.344	1.000
Age (years)	−0.025	0.867	1.000
Years of education	0.243	0.100	1.000
Number of risk factors	−0.018	0.902	1.000
AOO	0.027	0.859	1.000
DOI	0.035	0.816	1.000
H_Y OFF	−0.229	0.122	1.000
H_Y ON	0.022	0.881	1.000

MoCA TS: Montreal Cognitive Assessment Total Score, r_s_: Spearman coefficient, AOO: Age of Onset, DOI: Duration of Illness, H_Y: Hoehn and Yahr stage, which is a scale used to describe the severity and progression of Parkinson’s disease. Bonferroni-corrected p = min(1, raw p × 28).

**Table 7:** Relationship between serum granzyme B levels and different parameters in patient group (*n* = 47).

	No.	Serum Granzyme B Level (pg/mL)			Test of Sig.	*p*
		Min.–Max.	Mean ± SD	Median (IQR)		
Gender						
Male	36	792.3–4278.4	1744.5 ± 649.0	1693.9 (1330.7–1957.6)	U = 190.00	0.853
Female	11	897.9–2118.0	1646.0 ± 335.3	1617.1 (1530.1–1804.6)		
Residency						
Rural	21	868.9–4278.4	1786.6 ± 690.2	1707.3 (1531.5–1927.3)	U = 248.00	0.593
Urban	26	792.3–2943.3	1668.7 ± 500.2	1641.8 (1389.7–1938.0)		
Education						
Illiterate	4	1239.5–1531.5	1388.0 ± 164.1	1390.6 (1246.0–1530.1)	H = 9.504	0.091
Read & Write	4	897.9–1863.5	1329.3 ± 485.4	1277.9 (918.3–1740.3)		
Primary	9	792.3–2321.4	1650.7 ± 413.3	1716.1 (1464.3–1859.5)		
Preparatory	9	1150.8–2179.3	1551.7 ± 344.1	1476.3 (1334.2–1666.4)		
High school	12	975.7–2750.5	1862.25 ± 423.5	1884.7 (1664.7–2045.35)		
University graduated	9	868.9–4278.4	2096.5 ± 1000.2	1865.4 (1562.70–2118.0)		
Functioning						
Non-functioning	8	897.9–2113.5	1544.6 ± 425.6	1633.1 (1235.1–1804.6)	H = 1.026	0.599
Functioning	19	792.3–4278.4	1759.6 ± 765.5	1576.5 (1427.0–1952.25)		
Functioning & Retired	20	975.7–2943.3	1755.9 ± 448.9	1779.1 (1389.5–1959.15)		
Smoking						
Non-smoker	19	897.9–2943.3	1785.0 ± 461.6	1649.0 (1547.1–1901.7)	H = 7.202 *	0.027 *
Smoker	16	1211.5–4278.4	1905.0 ± 713.6	1852.8 (1503.8–2041.1)		
Ex-Smoker	11	792.3–2321.4	1349.6 ± 483.0	1334.2 (957.2–1546.8)		
Diabetes Mellitus						
No	32	792.3–2943.3	1651.52 ± 410.1	1657.7 (1417.2–1865.4)	U = 217.00	0.600
Yes	15	868.9–4278.4	1870.50 ± 855.3	1680.5 (1358.4–2197.1)		
Hypertension						
No	26	792.3–4278.4	1849.3 ± 691.7	1698.3 (1528.7–2113.5)	U = 210.00	0.178
Yes	21	868.9–2179.3	1563.1 ± 389.4	1571.25 (1327.2–1865.4)		
Hepatitis C virus						
No	35	792.3–4278.4	1718.68 ± 651.9	1649.0 (1417.2–1898.75)	U = 185.00	0.542
Yes	12	1150.8–2321.4	1729.36 ± 368.7	1864.4(1399.25–1952.25)		
Isolated Systolic Hypertension						
No	43	792.3–4278.4	1728.7 ± 610.5	1666.4 (1426.98–1957.6)	U = 83.00	0.927
Yes	4	1334.2–1927.3	1642.9 ± 297.2	1655.1 (1389.5–1896.35)		
Hyperlipidemia						
No	36	792.3–4278.4	1735.55 ± 634.4	1633.0 (1361.9–1959.15)	U = 177.00	0.611
Yes	11	868.9–2214.8	1675.1 ± 427.8	1859.5 (1618.0–1896.3)		
Consanguinity						
No	35	792.3–4278.4	1715.0 ± 611.7	1666.4 (1454.5–1865.4)	U = 198.00	0.770
Yes	12	938.7–2750.5	1740.0 ± 541.0	1754.2 (1269.35–2109.25)		
Family history of neuropsychiatric illness						
No	37	792.3–2943.3	1700.2 ± 460.6	1666.4 (1444.72–1938.0)	U = 176.00	0.828
Yes	10	868.9–4278.4	1799.8 ± 956.3	1682.5 (1211.5–1927.3)		
Family history of Parkinson’s						
No	35	897.9–2943.3	1672.8 ± 396.8	1648.9 (1454.5–1865.4)	U = 191.00	0.643
Yes	12	792.3–4278.4	1863.1 ± 966.2	1783.4 (1095.6–2150.85)		
Levodopa						
No	23	792.3–4278.4	1763.0 ± 644.7	1648.9 (1503.9–1865.4)	U = 272.00	0.932
Yes	24	868.9–2943.3	1681.55 ± 540.7	1725.6 (1283.35–1959.15)		
DOPA agonists						
No	34	792.3–2943.3	1642.5 ± 492.3	1657.7 (1334.2–1865.4)	U = 175.00	0.274
Yes	13	1239.5–4278.4	1927.7 ± 773.8	1743.9 (1576.5–1980.3)		
Amantadine						
No	27	792.3–4278.4	1753.6 ± 649.0	1707.3 (1454.5–1978.75)	U = 244.00	0.576
Yes	20	897.9–2943.3	1678.0 ± 509.3	1633.0 (1361.9–1853.75)		
Other treat						
No	14	792.3–2214.8	1650.7 ± 397.3	1610.1 (1464.3–1980.3)	U = 222.00	0.834
Yes	33	868.9–4278.4	1751.4 ± 656.6	1707.3 (1389.7–1927.3)		

IQR: Interquartile range; SD: Standard deviation; U: Mann–Whitney test; *p*: *p*-value for relation between serum granzyme B level and different parameters; *: Statistically significant at *p* ≤ 0.05.

## Data Availability

Data are available in the manuscript.

## Abbreviations

The following abbreviations are used in this manuscript:

PD	Parkinson’s disease
GrB	Granzyme B
DN	Dopaminergic neurons
PAR-1	Protease-activated receptor-1
H_Y	Hoehn and Yahr
MoCA	Montreal Cognitive Assessment
MoCA-TS	Montreal Cognitive Assessment Total Score
IQR	Interquartile range
DOI	Duration of illness
AOO	Age of onset
DM	Diabetes Mellitus
HTN	Hypertension
HCV	Hepatitis C virus
SD	Standard deviation
FE	Fisher Exact test
MC	Monte Carlo test
